# FliW and CsrA Govern Flagellin (FliC) Synthesis and Play Pleiotropic Roles in Virulence and Physiology of *Clostridioides difficile* R20291

**DOI:** 10.3389/fmicb.2021.735616

**Published:** 2021-10-05

**Authors:** Duolong Zhu, Shaohui Wang, Xingmin Sun

**Affiliations:** Department of Molecular Medicine, Morsani College of Medicine, University of South Florida, Tampa, FL, United States

**Keywords:** *Clostridioides difficile*, FliW, FliC, CsrA, R20291, virulence

## Abstract

*Clostridioides difficile* flagellin FliC is associated with toxin gene expression, bacterial colonization, and virulence, and is also involved in pleiotropic gene regulation during *in vivo* infection. However, how *fliC* expression is regulated in *C. difficile* remains unclear. In *Bacillus subtilis*, flagellin homeostasis and motility are coregulated by flagellar assembly factor (FliW), flagellin Hag (FliC homolog), and Carbon storage regulator A (CsrA), which is referred to as partner-switching mechanism “FliW-CsrA-Hag.” In this study, we characterized FliW and CsrA functions by deleting or overexpressing *fliW*, *csrA*, and *fliW*-*csrA* in *C. difficile* R20291. We showed that *fliW* deletion, *csrA* overexpression in R20291, and *csrA* complementation in R20291ΔWA (*fliW*-*csrA* codeletion mutant) dramatically decreased FliC production, but not *fliC* gene transcription. Suppression of *fliC* translation by *csrA* overexpression can be relieved mostly when *fliW* was coexpressed, and no significant difference in FliC production was detected when only *fliW* was complemented in R20291ΔWA. Further, loss of *fliW* led to increased biofilm formation, cell adhesion, toxin production, and pathogenicity in a mouse model of *C. difficile* infection (CDI), while *fliW*-*csrA* codeletion decreased toxin production and mortality *in vivo*. Our data suggest that CsrA negatively modulates *fliC* expression and FliW indirectly affects *fliC* expression through inhibition of CsrA post-transcriptional regulation. In light of “FliW-CsrA-Hag” switch coregulation mechanism reported in *B. subtilis*, our data also suggest that “FliW-CsrA-*fliC*/FliC” can regulate many facets of *C. difficile* R20291 pathogenicity. These findings further aid us in understanding the virulence regulation in *C. difficile*.

## Introduction

*Clostridioides difficile* (Lawson et al., 2016; Oren and Garrity, 2018) is a Gram-positive, spore-forming, toxin-producing, anaerobic bacterium that is a leading cause of nosocomial antibiotic-associated diarrhea in the developed countries (Sebaihia et al., 2006). *Clostridioides difficile* infection (CDI) can result in a spectrum of symptoms, ranging from mild diarrhea to pseudomembranous colitis and potential death (Lessa et al., 2012). *Clostridioides difficile* has many virulence factors, among which toxin A (TcdA) and toxin B (TcdB) are the major ones (Lyras et al., 2009; Kuehne et al., 2010). These toxins can disrupt the actin cytoskeleton of intestinal cells through glucosylation of the Rho family of GTPases, and induce mucosal inflammation and symptoms associated with CDI (Peniche et al., 2013).

CsrA, the carbon storage regulator A, has been reported to control various physiological processes, such as flagella synthesis, virulence, central carbon metabolism, quorum sensing, motility, and biofilm formation in pathogens including *Pseudomonas aeruginosa*, *Pseudomonas syringae*, *Borrelia burgdorferi*, *Salmonella typhimurium*, and *Proteus mirabilis* (Sabnis et al., 1995; Pessi et al., 2001; Lawhon et al., 2003; Lucchetti-Miganeh et al., 2008; Timmermans and Van Melderen, 2010; Karna et al., 2011; Morris et al., 2013; Ferreiro et al., 2018). Recently, the role of CsrA on carbon metabolism and virulence-associated processes in *C. difficile* 630Δ*erm* was analyzed by overexpressing the *csrA* gene (Gu et al., 2018). Authors showed that the *csrA* overexpression resulted in flagella defect, poor motility, and induced carbon metabolism change. Oppositely, toxin production and cell adherence increased in the *csrA* overexpression strain. CsrA is a widely distributed RNA binding protein that post-transcriptionally modulates gene expression through regulating mRNA stability and/or translation initiation of target mRNA (Romeo et al., 1993; Liu et al., 1995; Timmermans and Van Melderen, 2010). It typically binds to multiple specific sites that are located nearby or overlapping the cognate Shine–Dalgarno (SD) sequence in the target transcripts (Sorger-Domenigg et al., 2007; Yakhnin et al., 2007). The roles of CsrA in *Bacillus subtilis* have been well-studied (Yakhnin et al., 2007; Mukherjee et al., 2011; Oshiro et al., 2019). Flagellin Hag (FliC homolog), a main structure flagellar component, has been reported to be regulated by CsrA in *B. subtilis*. Yakhnin et al. (2007) first reported that CsrA in *B. subtilis* can regulate translation initiation of Hag by preventing ribosome binding to the *hag* transcript. Mukherjee et al. (2011) elucidated that the interaction between CsrA and FliW could govern flagellin homeostasis and checkpoint on flagellar morphogenesis in *B. subtilis*. FliW, the first protein antagonist of CsrA activity, was also identified and characterized in *B. subtilis*. They elegantly demonstrated a novel regulation system “a partner-switching mechanism” (Hag-FliW-CsrA) on flagellin synthesis in *B. subtilis*. Briefly, following the flagellar assembly checkpoint of hook completion, FliW was released from a FliW-Hag complex. Afterward, FliW binds to CsrA which will relieve CsrA-mediated *hag* translation repression for flagellin synthesis concurrent with filament assembly. Thus, flagellin homeostasis restricts its own expression on the translational level. Results also suggested that CsrA has an ancestral role in flagella assembly and has evolved to coregulate multiple cellular processes with motility. Oshiro et al. (2019) further quantitated the interactions in the Hag-FliW-CsrA system. They found that Hag-FliW-CsrA^dimer^ functions at nearly 1:1:1 stoichiometry in *B. subtilis*. The Hag-FliW-CsrA^dimer^ system is hypersensitive to the cytoplasmic Hag concentration and is robust to perturbation.

*Clostridioides difficile* flagellin gene *fliC* is associated with toxin gene expression, bacterial colonization, and virulence, and is responsible for pleiotropic gene regulation during *in vivo* infection (Tasteyre et al., 2001; Aubry et al., 2012; Baban et al., 2013; Barketi-Klai et al., 2014; Stevenson et al., 2015). The delicate regulations among *fliC* gene expression, toxin production, bacterial motility, colonization, and pathogenicity in *C. difficile* are indicated. Though the important roles of CsrA in flagellin synthesis and flagellin homeostasis have been studied in other bacteria (Yakhnin et al., 2007; Mukherjee et al., 2011; Oshiro et al., 2019), the regulation of FliW, CsrA, and FliC and the function of *fliW* in *C. difficile* remain unclear.

In this communication, we aimed to study the involvement of FliW and CsrA in *fliC* expression and *C. difficile* virulence and physiology by constructing and analyzing *fliW* and *fliW*-*csrA* deletion mutants of *C. difficile* R20291. We evaluated these mutants in the expression of *fliC*, motility, adhesion, biofilm formation, toxin production, sporulation, germination, and pathogenicity in a mouse model of CDI.

## Materials and Methods

### Bacteria, Plasmids, and Culture Conditions

Table 1 lists the strains and plasmids used in this study. *Clostridioides difficile* strains were cultured in BHIS media (brain heart infusion broth supplemented with 0.5% yeast extract and 0.1% L-cysteine, and 1.5% agar for agar plates) at 37°C in an anaerobic chamber (90% N_2_, 5% H_2_, and 5% CO_2_). For spores preparation, *C. difficile* strains were cultured in Clospore media and purified as described earlier (Perez et al., 2011). *Escherichia coli* DH5α and *E. coli* HB101/pRK24 were grown aerobically at 37°C in LB media (1% tryptone, 0.5% yeast extract, and 1% NaCl). *Escherichia coli* DH5α was used as a cloning host, and *E. coli* HB101/pRK24 was used as a conjugation donor host. Antibiotics were added when needed for *E. coli*, 15μg/ml chloramphenicol; for *C. difficile*, 15μg/ml thiamphenicol, 250μg/ml D-cycloserine, 50μg/ml kanamycin, 8μg/ml cefoxitin, and 500ng/ml anhydrotetracycline.

**Table 1:** Bacteria and plasmids utilized in this study.

Strains or plasmids	Genotype or phenotype	Reference
Strains		
*E. coli* DH5α	Cloning host	NEB
*E. coli* HB101/pRK24	Conjugation donor	Williams et al., 1990
*C. difficile* R20291	Clinical isolate; ribotype 027	Stabler et al., 2009
R20291ΔW	R20291 deleted *fliW* gene	This work
R20291ΔWA	R20291 deleted *fliW*-*csrA* genes	This work
R20291-E	R20291 containing blank plasmid pMTL84153	This work
R20291ΔW-E	R20291ΔW containing blank plasmid pMTL84153	This work
R20291ΔWA-E	R20291ΔWA containing blank plasmid pMTL84153	This work
R20291ΔW-W	R20291ΔW complemented with pMTL84153-*fliW*	This work
R20291ΔWA-WA	R20291ΔWA complemented with pMTL84153-*fliW*-*csrA*	This work
R20291ΔWA-W	R20291ΔWA complemented with pMTL84153-*fliW*	This work
R20291ΔWA-A	R20291ΔWA complemented with pMTL84153-*csrA*	This work
R20291-W	R20291 containing pMTL84153-*fliW*	This work
R20291-A	R20291 containing pMTL84153-*csrA*	This work
R20291-WA	R20291 containing pMTL84153-*fliW*-*csrA*	This work
Plasmids		
pDL1	AsCpfI based gene deletion plasmid	This work
pUC57-PsRNA	sRNA promoter template	This work
pDL1-*fliW*	*fliW* gene deletion plasmid	This work
pDL1-*csrA*	*csrA* gene deletion plasmid	This work
pDL1-*fliW-csrA*	*fliW-csrA* gene deletion plasmid	This work
pMTL84153	Complementation plasmid	Heap et al., 2009
pMTL84153-*fliW*-*csrA*	pMTL84153 containing *fliW*-*crsA* genes	This work
pMTL84153-*fliW*	pMTL84153 containing *fliW* gene	This work
pMTL84153-*csrA*	pMTL84153 containing *crsA* gene	This work

### DNA Manipulations and Chemicals

DNA manipulations were carried out according to standard techniques (Chong, 2001). Plasmids were conjugated into *C. difficile* as described earlier (Heap et al., 2010). The DNA markers, protein markers, PCR product purification kit, DNA gel extraction kit, restriction enzymes, cDNA synthesis kit, and SYBR Green RT-qPCR kit were purchased from ThermoFisher Scientific (Waltham, United States). PCRs were performed with the high-fidelity DNA polymerase NEB Q5 Master Mix, and PCR products were assembled into target plasmids with NEBuilder HIFI DNA Assembly Master Mix (New England, United Kingdom). Primers (Supplementary Table 1) were purchased from IDT (Coralville, United States). All chemicals were purchased from Sigma-Aldrich (St. Louis, United States) unless those stated otherwise.

### Gene Deletion, Complementation, and Overexpression in R20291

Gene edit plasmid pDL-1 containing Cas12a (AsCpfI) under the control of tetracycline-inducing promoter was constructed and used for *C. difficile* gene deletion according to a previous report (Hong et al., 2018). The target sgRNA was designed with an available website tool,^1^ and the off-target prediction was analyzed on the Cas-OFFinder website.^2^ The sgRNA, up- and down-homologous arms, were assembled into pDL-1. Two target sgRNAs for one gene deletion were selected and used for gene deletion plasmid construction in *C. difficile*, respectively. Briefly, the gene deletion plasmid was constructed in the cloning host *E. coli* DH5α and was transformed into the donor host *E. coli* HB101/pRK24, and subsequently was conjugated into R20291. Potential successful transconjugants were selected with selective antibiotic BHIS-TKC plates (15μg/ml thiamphenicol, 50μg/ml kanamycin, and 8μg/ml cefoxitin). The transconjugants were cultured in BHIS-Tm broth (15μg/ml thiamphenicol) to log phase, then the subsequent cultures were diluted with PBS serially and plated on the inducing plates (BHIS-Tm-ATc: 15μg/ml thiamphenicol and 500ng/ml anhydrotetracycline). The plates were incubated at 37°C in the anaerobic chamber for 24–48h, then 20–40 colonies were used as templates for colony PCR test with check primers for correct gene deletion colony isolation. The correct gene deletion colony was sub-cultured into BHIS broth without antibiotics and was passaged several times to cure the deletion plasmid, and then the cultures were plated on BHIS plates and subsequent colonies were replica plated on BHIS-Tm plates to isolate pure gene deletion mutants. The genome of R20291Δ*fliW* (referred hereafter as R20291ΔW) and R20291Δ*fliW*-*csrA* (referred hereafter as R20291ΔWA) were isolated and used as templates for the PCR test with check primers, and the PCR products were sequenced to confirm the correct gene deletion.

The *fliW* (396bp; primers 3-F/R), *csrA* (213bp; primers 4-F/R), and *fliW*-*csrA* (599bp; primers 5-F/R) genes were amplified and assembled into *Sac*I-*Bam*HI digested pMTL84153 plasmid, yielding the complementation plasmid pMTL84153-*fliW*, pMTL84153-*csrA*, and pMTL84153-*fliW*-*csrA*, and were subsequently conjugated into R20291ΔWA, R20291ΔW, and R20291 yielding complementation strain R20291ΔWA/pMTL84153-*fliW* (referred as R20291ΔWA-W), R20291ΔWA/pMTL84153-*csrA* (R20291ΔWA-A), R20291ΔWA/pMTL84153-*fliW*-*csrA* (R20291ΔWA-WA), and R20291ΔW/pMTL84153-*fliW* (R20291ΔW-W), and overexpression strain R20291/pMTL84153-*fliW* (R20291-W), R20291/pMTL84153-*csrA* (R20291-A), and R20291/pMTL84153- *fliW*-*csrA* (R20291-WA).

### Growth Profile, Motility, and Biofilm Assay

*Clostridioides difficile* strains were incubated to an optical density of OD_600_ of 0.8 in BHIS media and were diluted to an OD_600_ of 0.2. Then, 1% of the culture was inoculated into fresh BHIS, followed by measuring OD_600_ for 32h.

To examine the effect of *fliW* and *fliW*-*csrA* deletion on *C. difficile* motility, R20291, R20291ΔWA, and R20291ΔW were cultured to an OD_600_ of 0.8. For swimming analysis, 2μl of *C. difficile* culture was penetrated into soft BHIS agar (0.175%) plates, meanwhile, 2μl of culture was dropped onto 0.3% BHIS agar plates for swarming analysis. The swimming assay plates were incubated for 24h, and the swarming plates were incubated for 48h, respectively.

For biofilm formation analysis, wild-type and mutant strains were cultured to an OD_600_ of 0.8, and 1% of *C. difficile* cultures were inoculated into reinforced clostridial medium (RCM) with eight-well repeats in a 96-well plate and incubated in the anaerobic chamber at 37°C for 48h. Biofilm formation was analyzed by crystal violet dye. Briefly, *C. difficile* cultures were removed by pipette carefully. Then, 100μl of 2.5% glutaraldehyde was added into the well to fix the bottom biofilm, and the plate was kept at room temperature for 30min. Next, the wells were washed with PBS three times and dyed with 0.25% (w/v) crystal violet for 10min. The crystal violet solution was removed, and the wells were washed five times with PBS, followed by the addition of acetone into wells to dissolve the crystal violet of the cells. The dissolved solution was further diluted with ethanol 2–4 times, and biomass was determined at OD_570_.

### Adherence of *C. difficile* Vegetative Cells to HCT-8 Cells

*Clostridioides difficile* adhesion ability was evaluated with HCT-8 cells (ATCC CCL-244; Janvilisri et al., 2010). Briefly, HCT-8 cells were grown to 95% confluence (2×10^5^/well) in a 24-well plate and then moved into the anaerobic chamber, followed by infecting with 6×10^6^ of log phase of *C. difficile* vegetative cells at a multiplicity of infection (MOI) of 30:1. The plate was cultured at 37°C for 30min. After incubation, the infected cells were washed with 300μl of PBS three times, and then suspended in RPMI media with trypsin and plated on BHIS agar plates to enumerate the adhered *C. difficile* cells. The adhesion ability of *C. difficile* to HCT-8 cells was calculated as follows: CFU of adhered bacteria/total cell numbers.

To visualize the adherence of *C. difficile* to HCT-8 cells, *C. difficile* vegetative cells were labeled with the chemical 5(6)-CFDA (5- and−6)-Carboxyfluorescein diacetate (Fuller et al., 2000). Briefly, *C. difficile* strains were cultured to an OD_600_ of 0.8, then washed with PBS 3 times and resuspended in fresh BHIS supplemented with 50-mM 5(6)-CFDA, followed by incubation at 37°C for 30min in the anaerobic chamber. After post-incubation, the labeled *C. difficile* cells were collected and washed with PBS three times, and then resuspended in RPMI medium. Afterward, the labeled *C. difficile* cells were used for the infection experiment as described above. After 30-min post-infection, the fluorescence of each well was scanned by the multi-mode reader (excitation, 485nm; emission, 528nm), the relative fluorescence unit (RFU) was recorded as F0. Following, the plates were washed with PBS three times to remove unbound *C. difficile* cells, then the plates were scanned, and the RFU was recorded as F1. The adhesion ratio was calculated as follows: F1/F0. After scanning, the infected cell plates were further detected by the fluorescence microscope.

### *fliC* Expression Assay

For *fliC* transcription analysis, 2ml of 24-h post-inoculated *C. difficile* cultures were centrifuged at 4°C, 12,000×*g* for 5min, respectively. Then, the total RNA of different strains was extracted with TRIzol reagent. The transcription of *fliC* was measured by RT-qPCR with primers Q-*fliC*-F/R. All RT-qPCRs were repeated in triplicate, independently. Data were analyzed by the comparative CT (2^-∆∆CT^) method with 16s rRNA as a control.

To analyze the FliC protein level, *C. difficile* cell lysates from overnight cultures were used for Western blot analysis. Briefly, overnight *C. difficile* cultures were collected and washed three times with PBS and then resuspended in 5ml of distilled water. The suspensions were lysed with TissueLyser LT (Qiagen), followed centrifuged at 4°C, 25,000×*g* for 1h. The final pellets were resuspended in 30μl of PBS, and the total protein concentration was measured by using a BCA protein assay (Thermo Scientific, Suwanee, GA, United States). Protein extracts were subjected to 10% SDS-PAGE. Sigma A protein (SigA) was used as a loading control protein in SDS-PAGE (Mukherjee et al., 2013). FliC and SigA proteins on the gel were detected with anti-FliC and anti-SigA primary antibody (1:1,000, a generous gift from Dr. Daniel Kearns at Indiana University) and horseradish peroxidase-conjugated secondary antibody goat anti-mouse (Cat: ab97023, IgG, 1:3,000, Abcam, Cambridge, MA, United States) by Western blot, respectively. Anti-FliC antibody used in the Western blot analysis is an anti-FliCD serum, generated in the laboratory. FliCD is a fusion protein containing *C. difficile* FliC and FliD (Wang et al., 2018). The relative intensity of blot bands was analyzed by ImageJ software, and FliC relative intensity was normalized to SigA control.

### Toxin Expression Assay

To evaluate toxin expression in *C. difficile* strains, one single colony from each strain was inoculated into 25ml of BHIS and incubated in an anaerobic chamber at 37°C, and 10ml of *C. difficile* cultures from different strains were collected at 24- and 48-h post-incubation. The cultures were adjusted to the same OD_600_ value with fresh BHIS. Then, the collected *C. difficile* cultures were centrifuged at 4°C, 8,000×*g* for 15min, filtered with 0.22μm filters, and used for ELISA. Anti-TcdA (PCG4.1, Novus Biologicals, United States) and anti-TcdB (AI, Gene Tex, United States) were used as coating antibodies for ELISA, and HRP-Chicken anti-TcdA and HRP-Chicken anti-TcdB (Gallus Immunotech, United States) were used as detection antibodies.

For toxin transcription analysis, 2ml of 24- and 48-h post-inoculated *C. difficile* cultures were centrifuged at 4°C, 12,000×*g* for 5min, respectively. Next, the total RNA of different strains was extracted with TRIzol reagent. The transcription of *tcdA* and *tcdB* was measured by RT-qPCR with primers Q-*tcdA*-F/R and Q-*tcdB*-F/R, respectively. All RT-qPCRs were repeated in triplicate, independently. Data were analyzed by using the comparative CT (2^-∆∆CT^) method with 16s rRNA as a control.

### Germination and Sporulation Assay

*Clostridioides difficile* germination and sporulation analysis were conducted as reported earlier (Zhu et al., 2019). Briefly, for *C. difficile* sporulation analysis, *C. difficile* strains were cultured in Clospore media for 4days. Afterward, the CFU of cultures from 48 and 96h were counted on BHIS plates with 0.1% TA to detect sporulation ratio, respectively. The sporulation ratio was calculated as CFU (65°C heated, 20min)/CFU (no heated). For *C. difficile* germination analysis, *C. difficile* spores were collected from 2-week Clospore media-cultured bacteria and purified with sucrose gradient layer (50, 45, 35, 25, and 10%). The heated purified spores were diluted to an OD_600_ of 1.0 in the germination buffer [10mM Tris (pH 7.5), 150mM NaCl, 100mM glycine, and 10mM taurocholic acid (TA)] to detect the germination ratio. The value of OD_600_ was monitored immediately (0min, t_0_), and was detected once every 2min (t_x_) for 20min at 37°C. The germination ratio was calculated as OD_600_ (tx)/OD_600_ (T_0_). Spores in germination buffer without TA were used as the negative control.

### R20291, R20291ΔWA, and R20291ΔW Virulence in the Mouse Model of *C. difficile* Infection

C57BL/6 female mice (6weeks old) were ordered from Charles River Laboratories, Cambridge, MA. All studies were approved by the Institutional Animal Care and Use Committee of University of South Florida. The experimental design and antibiotic administration were conducted as described earlier (Sun et al., 2011). Briefly, 30 mice were divided into three groups in six cages. Group 1 mice were challenged with R20291 spores, group 2 mice with R20291ΔWA spores, and group 3 mice with R20291ΔW spores, respectively. Mice were given an orally administered antibiotic cocktail (kanamycin 0.4mg/ml, gentamicin 0.035mg/ml, colistin 0.042mg/ml, metronidazole 0.215mg/ml, and vancomycin 0.045mg/ml) in drinking water for 4days. After 4days of antibiotic treatment, all mice were given autoclaved water for 2days, followed by one dose of clindamycin (10mg/kg, intraperitoneal route) 24h before spores challenge (Day 0). After that mice were orally gavaged with 10^6^ spores and monitored daily for a week for changes in weight, diarrhea, and mortality. If body weight loss was equal to or greater than 20%, the mouse was euthanized and counted as a dead one. Mortality also included mice that were succumbed to disease. Diarrhea was defined as soft or watery feces. All survived mice were humanely euthanized on day 7 of post-*C. difficile* challenge.

### Enumeration of *C. difficile* Spores and Determination of Toxin Level in Feces

Fecal pellets from post-infection day 0 to day 7 were collected from each mouse and stored at −80°C. To enumerate *C. difficile* spores, feces were diluted with PBS at a final concentration of 0.1g/ml, followed by adding 900μl of absolute ethanol into 100μl of the fecal solution, and kept at room temperature for 1h to inactivate vegetative cells. Afterward, 200μl of vegetative cells inactivated fecal solution from the same group and the same day was mixed. Then, fecal samples were serially diluted and plated on BHIS-CCT plates (250μg/ml D-cycloserine, 8μg/ml cefoxitin, and 0.1% TA). After 48-h incubation, colonies were counted and expressed as CFU/g feces. To evaluate toxin tilter in feces, 0.1g/ml of the fecal solution was diluted two times with PBS, followed by examining TcdA and TcdB ELISA.

### Statistical Analysis

The reported experiments were conducted in independent biological triplicates, and each sample was additionally taken in technical triplicates. Animal survivals were analyzed by Kaplan–Meier survival analysis and compared by the log-rank test. One-way ANOVA with *post hoc* Tukey test was used for more than two groups’ comparison. Results were expressed as mean±SEM. Differences were considered statistically significant if *p*<0.05 (^*^).

## Results

### Highly Conserved *fliW* and *csrA* Genes in *C. difficile*

DNA and protein sequences of *fliW* and *csrA* from 10 *C. difficile* strains belonging to different ribotypes (RTs), including RT106, RT027, RT001, RT078, RT009, RT012, RT046, and RT017 were selected and aligned to those of R20291 (Table 2). We found that *fliW* and *csrA* genes are broadly found in *C. difficile* genomes, and both DNA and protein sequences of *fliW* and *csrA* are conserved across different *C. difficile* strains. These results motivated us to investigate the functions of *fliW* and *csrA* in *C. difficile*.

**Table 2:** Alignments of *fliW*-*csrA* DNA and protein sequences in *Clostridioides difficile* strains.

Strain	Sequence type (ribotype)	Genome accession	Identity (%)		
			DNA	Protein	
			*fliW*-*csrA*	FliW	CsrA
*C. difficile* DH	ST42 (RT106)	CP022524.1	100	100	100
*C. difficile* CD196	ST1 (RT027)	FN538970.1	100	100	100
*C. difficile* ATCC8689	ST3 (RT001)	CP011968.1	99.17	99.23	100
*C. difficile* TW11	ST11 (RT078)	CP035499.1	96.99	98.46	97.14
*C. difficile* M120	ST11 (RT078)	FN665653.1	96.99	98.46	97.14
*C. difficile* Z31	ST3 (RT009)	CP013196.1	88.98	83.85	92.86
*C. difficile* DSM27639	ST54 (RT012)	CP011847.1	88.81	83.85	92.86
*C. difficile* 630	ST54 (RT012)	CP010905.2	88.81	83.08	92.86
*C. difficile* CDT4	ST35 (RT046)	CP029152.1	88.65	83.08	92.86
*C. difficile* M68	ST37 (RT017)	FN668375.1	88.65	83.08	92.86

### Construction of *fliW* and *fliW*-*csrA* Deletion Mutants and Complementation Strains

**Figure 1:**
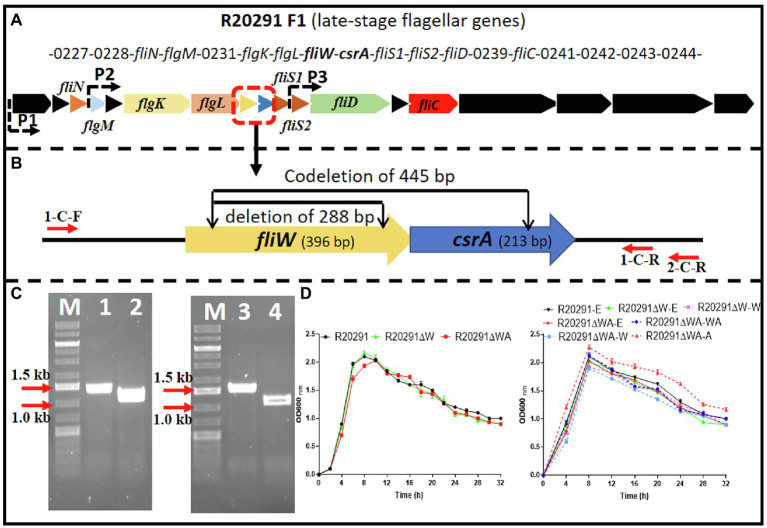
R20291 late-stage flagellar genes (F1) and *fliW* and *fliW*-*csrA* deletion. **(A)** Schematic representation of late-stage flagellar genes (F1). Dotted arrows (P1, P2, and P3) indicate the potential promoters in F1. **(B)** Deletion of *fliW* and *fliW*-*csrA* genes. 1-C-F/R were used to verify *fliW* deletion, and 1-C-F and 2-C-R were used to test *fliW*-*csrA* codeletion. **(C)** Verification of *fliW* and *fliW*-*csrA* deletions by PCR. M, DNA ladder; 1, R20291 genome as PCR template; 2, R20291∆W genome as PCR template; 3, R20291 genome as PCR template; and 4, R20291∆WA genome as PCR template. **(D)** Growth profile of parent strain and gene deletion mutants. Experiments were independently repeated thrice. Bars stand for mean±SEM. One-way ANOVA with *post hoc* Tukey test was used for statistical significance.

The *C. difficile* R20291 flagellar gene operon was analyzed through the *IMG*/*M* website,^3^ and the late-stage flagellar genes (F1) are drawn as Figure 1A (Stevenson et al., 2015). Among them, *fliW* and *csrA* genes have a 10bp overlap and were demonstrated as cotranscription by RT-PCR (Supplementary Figure 1).

To analyze the role of *fliW* and *csrA* in R20291 (NC_013316.1), CRISPR-AsCpfI-based plasmid pDL1 (pMTL82151-Ptet-AscpfI) was constructed for gene deletion in *C. difficile* (Zhu et al., 2021). pDL1-*fliW* and pDL1-*csrA* gene deletion plasmids were constructed, and the *fliW* gene (288bp deletion; R20291ΔW) was deleted successfully. However, after several trials, we could not get the *csrA* gene deletion mutant possibly due to its small size (213bp) or particularly unknown roles for R20291. We also tried to use Clostron and *pyrE* gene edit system to delete *csrA* gene, but failed to get the correct mutant. Therefore, we constructed *fliW*-*csrA* codeletion plasmid pDL1-*fliW*-*csrA*. Part of *fliW*-*csrA* (445bp deletion) gene was codeleted, and the plasmid curing mutant R20291ΔWA was obtained (Figure 1B,C). To study the role of *csrA* in R20291, the single gene complementation strain R20291ΔWA-W and R20291ΔWA-A were constructed. R20291, R20291-pMTL84153 (R20291-E), R20291ΔW-pMTL84153 (R20291ΔW-E), and R20291ΔWA-pMTL84153 (R20291ΔWA-E) were used as control strains when needed.

The effects of *fliW* and *fliW*-*csrA* deletion on R20291 growth were evaluated. Figure 1D shows that there was no significant difference in bacterial growth between parent strain and mutants in BHIS media.

### Effects of *fliW* and *fliW*-*csrA* Deletions on *C. difficile* Motility and Biofilm Formation

**Figure 2:**
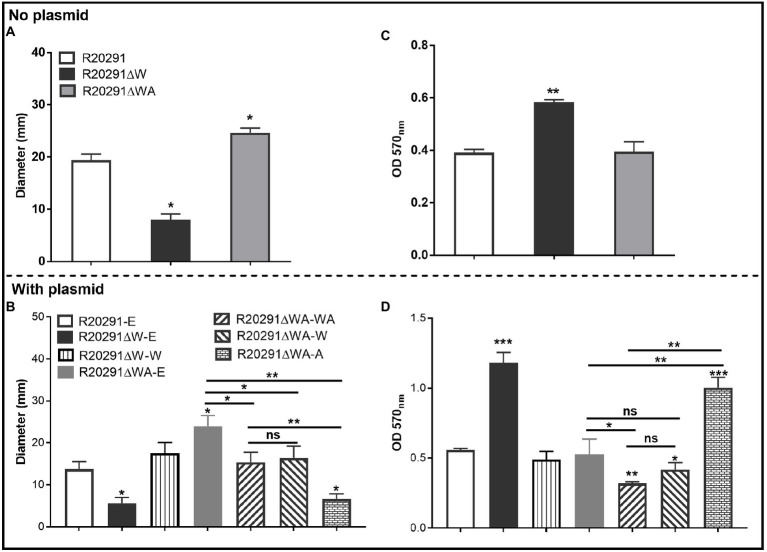
Motility and biofilm analysis. **(A,B)**: Halo diameter of motility (swimming analysis on 0.175% agar plate). **(C,D)**: Biofilm formation analysis. Bars stand for mean±SEM (^*^p<0.05, ^**^p<0.01, and ^***^p<0.001). One-way ANOVA with *post hoc* Tukey test was used for statistical significance. ^***^directly upon the column means the significant difference of the experimental strain compared to R20291 or R20291-E.

To characterize the effects of *fliW* and *fliW*-*csrA* deletions on *C. difficile* motility, swimming, and swarming motilities of R20291, R20291ΔWA, and R20291ΔW were first analyzed at 24 and 48-h post-inoculation (Figure 2A; Supplementary Figure 2), respectively. The diameter of the swimming halo of R20291ΔWA increased by 27.2% (*p*<0.05), while that of R20291ΔW decreased by 58.4% (*p*<0.05) compared to that of R20291. Next, we examined the motility of the complementation strains (Figure 2B; Supplementary Figure 2), and similar results were obtained among R20291-E, R20291ΔWA-E (with the swimming halo increased by 74.8%, *p*<0.05), and R20291ΔW-E (with the swimming halo decreased by 59.2%, *p*<0.05; Figure 2B). No significant difference was detected between complementation strain R20291ΔWA-WA, R20291ΔWA-W, R20291ΔW-W, and the parent strain R20291-E except R20291ΔWA-A which decreased by 52.0% (*p*<0.05) in swimming halo (Figure 2B). The swarming (48h) and swimming (24h) motilities analyzed on agar plates are shown in Supplementary Figure 2.

The effects of *fliW* and *fliW*-*csrA* deletions on *C. difficile* biofilm formation were also analyzed. In comparison with R20291, the biofilm formation of R20291ΔW increased by 49.5% (*p*<0.01), and no significant difference in biofilm formation was detected in R20291ΔWA (Figure 2C). The biofilm formation of R20291ΔW-E increased 112.3% (*p*<0.001) and R20291ΔWA-A increased by 79.9% (*p*<0.001) compared to R20291-E (Figure 2D). Meanwhile, the biofilm formation of R20291ΔWA-WA and R20291ΔWA-W decreased by 42.8% (*p*<0.01) and 25.2% (*p*<0.05), respectively.

Together, these data indicate that loss of FliW impairs *C. difficile* motility, and increases biofilm production. The decrease of motility and increase in biofilm production were also detected in R20291ΔWA-A, which was largely restored by coexpressing *fliW* with *csrA* in R20291ΔWA (Figures 2B,D), indicating that FliW could antagonize CsrA to regulate bacterial motility and biofilm production.

### Effects of *fliW* and *fliW*-*csrA* Deletions on Bacterial Adherence *in vitro*

**Figure 3:**
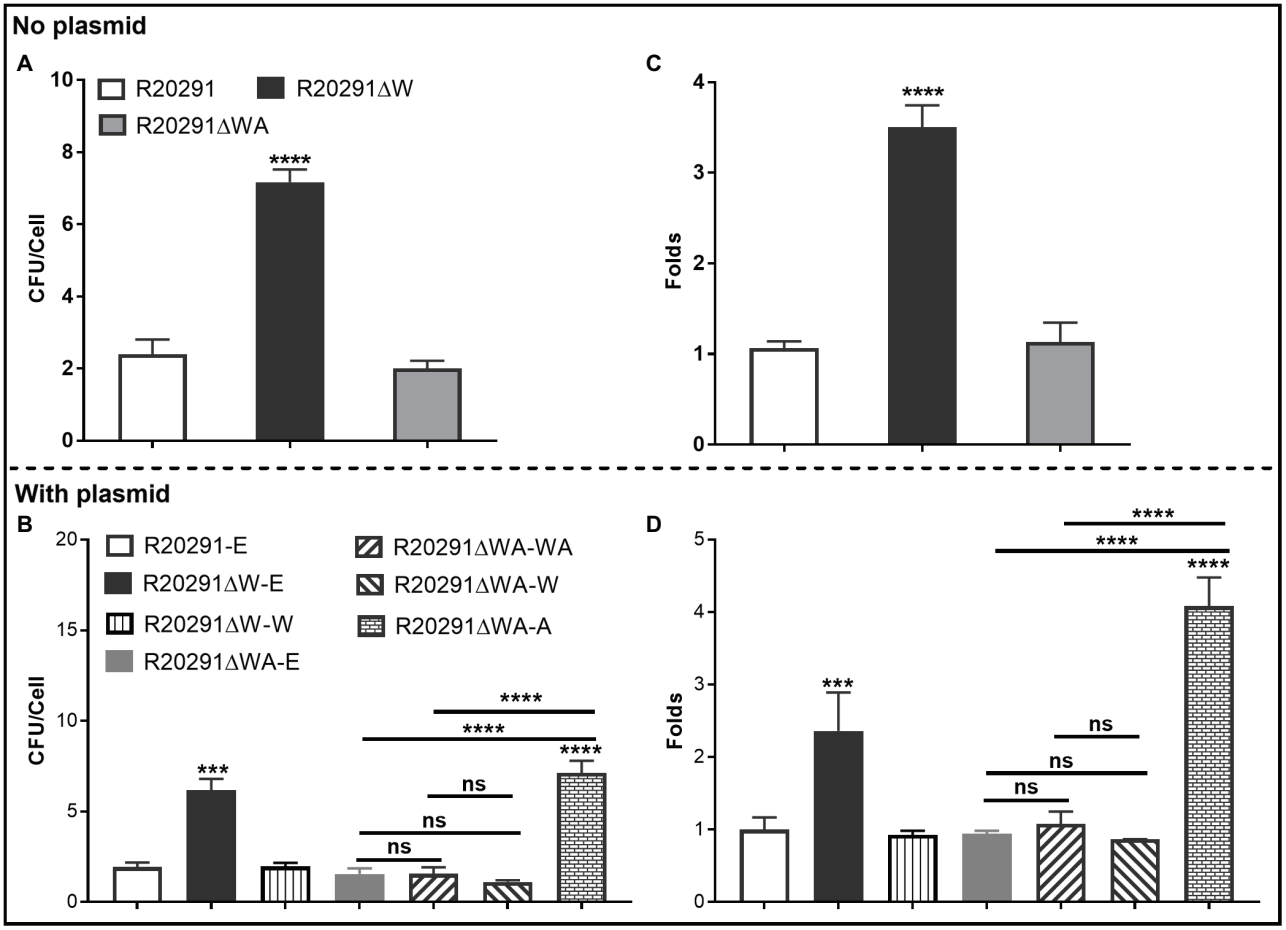
Adhesion analysis **(A,B)**: Adherence of *C. difficile* vegetative cells to HCT-8 cells *in vitro*. **(C,D)**: Adhesion analysis with 5(6)-CFDA dye. The fluorescence intensity was scanned by the multi-mode reader (excitation, 485nm; emission, 528nm). The original relative fluorescence unit (RFU) was recorded as F0, after PBS wash, the RFU was recorded as F1. The adhesion ratio was calculated as follows: F1/F0. Experiments were independently repeated thrice. Bars stand for mean±SEM (^*^p<0.05, ^**^p<0.01, ^***^p<0.001, and ^****^p<0.0001). One-way ANOVA with *post hoc* Tukey test was used for statistical significance. ^*^ directly upon the column means the significant difference of the experimental strain compared to R20291 or R20291-E.

The ability of *C. difficile* vegetative cells to adhere to HCT-8 cells *in vitro* was analyzed. Figure 3A shows that the mean adhesion number of R20291 was 2.40±0.70 bacteria/cell, while that of R20291ΔW was 7.17±0.61, which was 3.0-fold (*p* < 0.0001) of R20291. No significant difference was detected between R20291ΔWA and R20291. In the complementation strains, we detected a similar result which showed that the mean adhesion number of R20291ΔW-E (6.17±0.64) was 3.20-fold (*p* < 0.0001) of R20291-E (1.93±0.25; Figure 3B). The adhesion ability of complementation strains nearly recovered to that of wild-type strain except for R20291ΔWA-A (7.13±0.66, *p* < 0.0001) which was 3.69-fold of R20291-E in the mean adhesion number (Figure 3B).

To visualize the adhesion of *C. difficile* to HCT-8 cells, the *C. difficile* vegetative cells were labeled with the chemical 5(6)-CFDA. Figures 3C,D shows that the fluorescence intensity of R20291ΔW was 3.50-fold (*p* < 0.0001) of that in R20291, and the fluorescence intensity of R20291ΔW-E was 2.36-fold (*p* < 0.001), and R20291ΔWA-A was 4.08-fold (*p* < 0.0001) of that in R20291-E, respectively, which is consistent with the results shown in Figures 3A,B. Meanwhile, the adherence of *C. difficile* to HCT-8 cells was also visualized by fluorescence microscopy (Supplementary Figure 3).

Our data showed that FliW negatively affects bacterial adherence. CsrA complementation in R20291ΔWA increased adherence, while the phenotype change can be recovered partially when *fliW* was coexpressed with *csrA* in R20291ΔWA, suggesting that FliW could antagonize CsrA to regulate bacterial adherence. The results from bacterial adherence analysis were consistent with biofilm production analysis indicating the close relation between biofilm production and adherence in *C. difficile*.

### Effects of Deletion and Overexpression of *fliW* and *fliW*-*csrA* on *fliC* Expression

**Figure 4:**
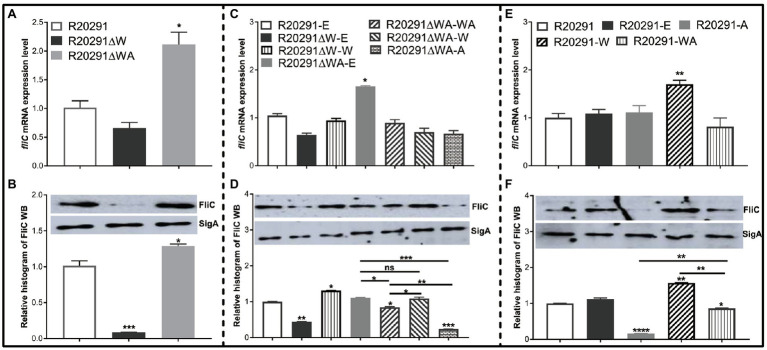
*fliC* expression analysis. **(A,C,E)** Analysis of *fliC* expression on transcription level. **(B,D,F)** Analysis of *fliC* expression on translation level by Western blot. SigA protein was used as a loading control. Experiments were independently repeated thrice. Bars stand for mean±SEM (^*^p<0.05, ^**^p<0.01, ^***^p<0.001, and ^****^p<0.0001). One-way ANOVA with *post hoc* Tukey test was used for statistical significance. ^****^ upon the column directly means the significant difference of experimental strain compared to R20291 or R20291-E.

In *B. subtilis*, FliW interacts with CsrA to regulate *hag* (a homolog of *fliC*) translation. We reasoned that FliW and CsrA would also regulate *fliC* expression in *C. difficile*. As shown in Figure 4A, the transcription of *fliC* in R20291ΔWA increased 1.12-fold (*p*<0.05), while the *fliW* deletion impaired the *fliC* transcription slightly while no significant difference. Figure 4B shows the production of FliC in R20291ΔW dramatically decreased (10.4-fold reduction, *p*<0.001), while that of R20291ΔWA increased significantly (increased by 27.5%, *p*<0.05). To further determine the role of the single-gene *csrA* on FliC synthesis, *csrA* and *fliW* were complemented into R20291ΔWA or overexpressed in R20291, respectively. Results showed that the significant difference of *fliC* transcription could only be detected in R20291ΔWA-E (increased by 32.3%, *p*<0.05; Figure 4C) and R20291-W (increased by 69.8%) compared to R20291-E (Figure 4E). Interestingly, the FliC production of R20291ΔWA-A decreased 3.2-fold (*p*<0.001) compared to that of R20291-E, while that of R20291ΔWA-WA only decreased by 14.3% (*p*<0.05), and no significant difference of FliC production in R20291ΔWA-W was detected (Figure 4D). As shown in Figures 4E,F, the *fliC* transcription of R20291-A was not affected compared to R20291-E, but the FliC production in R20291-A decreased 5.3-fold (*p*<0.0001). The decrease in FliC production in R20291-A can be partially recovered when *fliW* was coexpressed with *csrA* (R20291-WA decreased by 16.2%, *p*<0.05).

Collectively, our data indicate that CsrA negatively modulates *fliC* expression post-transcriptionally and FliW antagonizes CsrA to regulate *fliC* expression possibly through inhibiting CsrA-mediated negative post-transcriptional regulation.

### Effects of *fliW* and *fliW*-*csrA* Deletions on Toxin Expression

**Figure 5:**
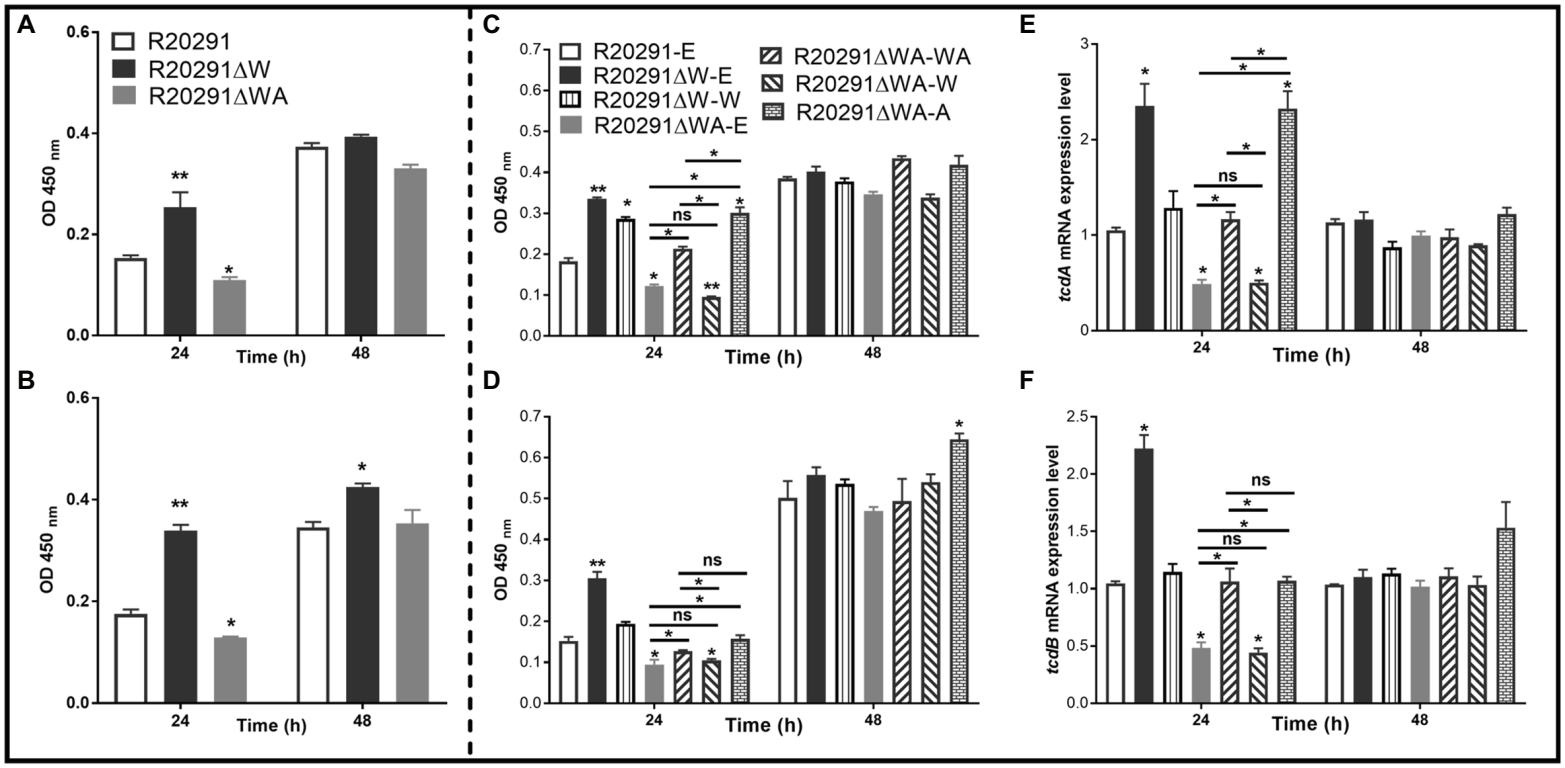
Toxin expression analysis. **(A)** TcdA concentration in the supernatants of R20291, R20291∆WA, and R20291∆W. **(B)** TcdB concentration in the supernatants of R20291, R20291∆WA, and R20291∆W. **(C)** TcdA concentration in the supernatants of parental and gene complementation strains. **(D)** TcdB concentration in the supernatants of parental and gene complementation strains. **(E)** Transcription of *tcdA* in the supernatants of parental and gene complementation strains. **(F)** Transcription of *tcdB* in the supernatants of parental and gene complementation strains. Experiments were independently repeated thrice. Bars stand for mean±SEM (^*^p<0.05, ^**^p<0.01). One-way ANOVA with *post hoc* Tukey test was used for statistical significance. ^**^ upon the column directly means the significant difference of experimental strain compared to R20291 or R20291-E.

It has been reported that the expression of *csrA* could affect toxin expression in *C. difficile* (Gu et al., 2018). To evaluate the effects of *fliW* and *fliW*-*csrA* deletions on toxin production, the supernatants of *C. difficile* cultures were collected at 24- and 48-h post-inoculation, and the toxin concentration was determined by ELISA. Figure 5A shows that the TcdA concentration of R20291ΔWA decreased by 28.6% (*p* < 0.05), while R20291ΔW increased by 65.1% (*p* < 0.01) compared to R20291 at 24-h post-inoculation. However, after 48-h incubation, no significant difference was detected. In Figure 5B, TcdB concentration of R20291ΔWA decreased by 26.4% (*p* < 0.05) at 24-h post-inoculation, while that of R20291ΔW increased by 93.6% (*p* < 0.01) at 24h and 33.0% (*p* < 0.05) at 48h. Similar results were also detected in the complementation strains group (Figures 5C,D). As shown in Figures 5C,D, after 24-h post-inoculation, TcdA (Figure 5C) concentration of R20291ΔWA-E and R20291ΔWA-W decreased by 33.0% (*p < 0.05) and 47.7% (*p* < 0.01), and TcdB (Figure 5D) concentration of R20291ΔWA-E and R20291ΔWA-W decreased by 37.9% (*p* < 0.05) and 31.3% (*p* < 0.05), respectively, while TcdA concentration of R20291ΔW-E, R20291ΔWA-A, and R20291ΔW-W increased by 83.1% (*p* < 0.01), 64.7% (*p* < 0.05), and 56.5% (*p* < 0.05), respectively. Meanwhile, TcdB concentration of R20291ΔW-E increased by 100.2% (*p* < 0.01). At 48-h post-inoculation, though no significant difference in TcdA production was detected among different *C. difficile* strains, TcdB concentration of R20291ΔWA-A increased by 28.5% (*p* < 0.05) compared to R20291-E.

To analyze the transcription of *tcdA* and *tcdB* in the complementation strains, RT-qPCR was performed. As shown in Figures 5E,F, the transcription of *tcdA* and *tcdB* of R20291ΔWA-E and R20291ΔWA-W decreased significantly (*p* < 0.05), while that of R20291ΔW-E increased significantly (*p* < 0.05). Interestingly, the *tcdA* transcription of R20291ΔWA-A also showed a significant increase (*p* < 0.05) compared to the wild-type strain. Our data indicate that FliW negatively regulates toxin expression, while CsrA plays a positive regulation role in toxin expression.

### Effects of *fliW* and *fliW*-*csrA* Deletions on Sporulation and Germination

To assay the sporulation ratio of *C. difficile* strains, R20291, R20291ΔWA, and R20291ΔW were cultured in Clospore media for 48 and 96h, respectively. Results (Supplementary Figure 4A) showed that no significant difference in the sporulation ratio was detected between the wild-type strain and the mutants. The germination ratio of *C. difficile* spores was evaluated as well. Purified spores of R20291, R20291ΔWA, and R20291ΔW were incubated in the germination buffer supplemented with taurocholic acid (TA). As shown in Supplementary Figure 4B, there was no significant difference in the germination ratio between the wild-type strain and the mutants.

### Evaluation of *fliW* and *fliW*-*csrA* Deletions on Bacterial Virulence in the Mouse Model of CDI

**Figure 6:**
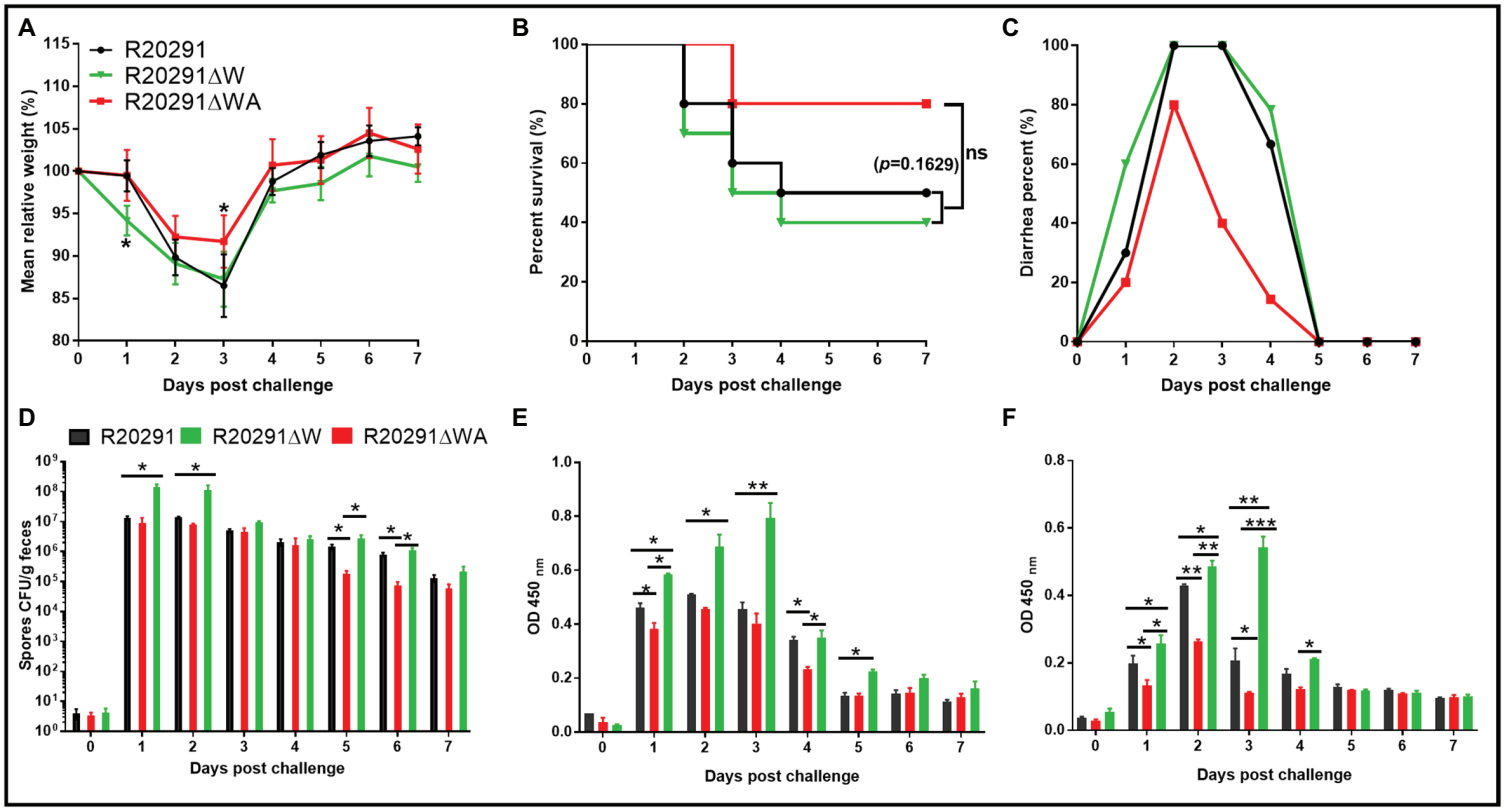
Effects of *fliW* and *fliW-csrA* deletion on *C. difficile* virulence in mice. **(A)** Mean relative weight changes. **(B)** Survival curve. **(C)** Diarrhea percentage. **(D)** *Clostridioides difficile* in feces. **(E)** TcdA titer of fecal sample. **(F)** TcdB titer of fecal sample. Bars stand for mean±SEM (^*^p<0.05, ^**^p<0.01, ^***^p<0.001). One-way ANOVA with *post hoc* Tukey test was used for statistical significance. Animal survivals were analyzed by Kaplan–Meier survival analysis with a log-rank test of significance.

To evaluate the effects of *fliW* and *fliW*-*csrA* deletions on *C. difficile* virulence *in vivo*, the mouse model of CDI was used. Thirty mice (*n*=10 per group) were orally challenged with R20291, R20291ΔWA, or R20291ΔW spores (1×10^6^ spores/mouse) after antibiotic treatment. As shown in Figure 6A, the R20291ΔW infection group lost more weight at post-challenge days 1 (*p* < 0.05), and the R20291ΔWA infection group lost less weight at post-challenge days 3 (*p* < 0.05) compared to the R20291 infection group. Figure 6B shows that 60% of mice succumbed to severe disease within 4days in the R20291ΔW infection group and 20% in the R20291ΔWA infection group compared to 50% mortality in the R20291 infection group (no significant difference with log-rank analysis, *p*=0.1629). Meanwhile, 100% of mice developed diarrhea in both the R20291ΔW and R20291 infection groups vs. 80% in the R20291ΔWA infection group at post-challenge days 2 (Figure 6C). As shown in Figure 6D, the spores CFU of the R20291ΔW infection group increased in the fecal shedding samples at post-challenge days 1 and 2 (*p* < 0.05), while the spores CFU of the R20291ΔWA infection group decreased at post-challenge days 1, 5, and 6 (*p* < 0.05) compared to the R20291 infection group. Interestingly, while we did not detect significant differences in bacterial growth, germination, and sporulation between the wild-type strain and mutants, the spore numbers from different infection groups were different (Figure 6D). This kind of difference implied that the culture media we used *in vitro* cannot simulate the complicated intestine environment well, which can lead to the different outcomes in bacterial physiology between *in vitro* and *in vivo* analysis. CsrA, as a carbon storage regulator, its regulation on carbon metabolization, and other potential roles in the complicated gut environment *in vivo* remain to be further studied.

To evaluate the toxin level in the gut, the concentrations of TcdA and TcdB in the feces were measured by ELISA. In comparison with the R20291 infection group, the TcdA of the R20291ΔW infection group increased significantly at post-challenge days 1 (*p* < 0.05), 2 (*p* < 0.05), 3 (*p* < 0.01), and 5 (*p* < 0.05; Figure 6E), while the TcdA of the R20291ΔWA infection group decreased significantly at post-challenge days 1 (*p* < 0.05) and 4 (*p* < 0.05; Figure 6E). As shown in Figure 6F, the TcdB concentration of the R20291ΔWA infection group decreased significantly at post-challenge days 1 (*p* < 0.05), 2 (*p* < 0.05), and 3 (*p* < 0.05), and that of the R20291ΔW increased significantly at post-challenge days 1 (*p* < 0.05), 2 (*p* < 0.01), and 3 (*p* < 0.01). Taken together, our results indicate that the FliW defect increases R20291 pathogenicity *in vivo*, while the *fliW*-*csrA* codeletion impairs R20291 pathogenicity.

## Discussion

In this study, we sought to characterize the impacts of FliW, CsrA, and FliC on *C. difficile* pathogenicity. Our data suggest that CsrA negatively modulates *fliC* expression post-transcriptionally, and FliW affects *fliC* expression possibly through inhibiting CsrA-mediated negative post-transcriptional regulation. Our data also indicate that FliW negatively affects *C. difficile* pathogenicity possibly by antagonizing CsrA *in vivo*. Based on our current pleiotropic phenotype analysis, a similar partner-switching mechanism “FliW-CsrA-*fliC*/FliC” (FliC binds FliW, FliW binds CsrA, and CsrA regulates *fliC* translation by binding to 5′ untranslated *region* of *fliC* transcripts) is predicted in *C. difficile*, though more direct experimental data are needed to uncover the molecular interactions of CsrA, FliW, and *fliC*/FliC in *C. difficile* (Supplementary Figure 5).

It has been reported that overexpression of the *csrA* gene could result in flagella defects, poor motility, and increased toxin production and adhesion in *C. difficile* 630Δerm (Gu et al., 2018). In our study, we found that CsrA and FliW widely exist in *C. difficile* (Table 2), even in the *C. difficile* strains without flagella like *C. difficile* M120 (Stabler et al., 2009), indicating a potentially important role of FliW-CsrA in *C. difficile*. Interestingly, while there are no flagella in *C. difficile* M120, six flagellar structure genes (*fliS*, *fliN*, *flgK*, *flgL*, *fliC*, and *fliD*) are still found in the genome, which inspired us to explore the potential roles of *fliW*, *csrA*, and *fliC* in *C. difficile* by deleting or overexpressing *fliW*, *csrA*, and *fliW*-*csrA* genes. However, after several trials with different gene edit methods in *C. difficile*, we could not get the *csrA* gene deletion mutant possibly due to its small size. This result motivated us to construct *fliW*-*csrA* double deletion mutant. While we did not get the single *csrA* gene deletion, we complemented the single *fliW* gene in the *fliW*-*csrA* double deletion mutant for simulation of the *csrA* deletion effects. The important roles of CsrA in flagellin synthesis and flagellin homeostasis have been reported (Yakhnin et al., 2007; Mukherjee et al., 2011; Gu et al., 2018; Oshiro et al., 2019). A previous study had shown that the overexpression of the *csrA* gene can cause a dramatic motility reduction and a significant Hag decrease in *B. subtilis* (Yakhnin et al., 2007). FliW (the first protein regulator of CsrA activity) deletion abolished the *B. subtilis* swarming and swimming motility and decreased the number of flagella and flagellar length (Mukherjee et al., 2011, 2016). In this study, we obtained similar results that FliW defect impaired R20291 motility significantly (Figure 2A) and increased biofilm formation (Figures 2C,D). Interestingly, the *csrA* gene complementation in R20291ΔWA dramatically suppressed bacterial motility and showed a similar result to R20291ΔW, indicating that CsrA can suppress *C. difficile* motility and increase biofilm production, while FliW antagonizes *csrA* to regulate bacteria motility and biofilm formation indirectly.

The partner-switching mechanism “Hag-FliW-CsrA” on flagellin synthesis was elucidated in *B. subtilis*, and the intracellular concentration of the flagellar filament protein Hag is restricted tightly by the Hag-FliW-CsrA system (Mukherjee et al., 2011). To investigate whether FliW and CrsA coregulate the *fliC* expression in *C. difficile*, we evaluated both the transcriptional and translational expression level of *fliC* gene. Our data (Figure 4) showed that the *fliW* deletion resulted in a 10.4-fold decrease in FliC accumulation, while the *fliW*-*csrA* codeletion increased FliC production, indicating that CsrA could suppress the *fliC* translation and FliW antagonizes CsrA to regulate FliC production. In *csrA*, *fliW*, and *fliW*-*csrA* overexpression experimental groups, we found that the *csrA* overexpression dramatically decreased FliC production (5.3-fold reduction) and the reduction in FliC production in R20291-A can be partially recovered when *fliW*-*csrA* was coexpressed. The FliW complementation in R20291ΔWA did not affect FliC production, but the *fliW* overexpression in R20291 increased FliC production. Taken together, our data suggest that CsrA negatively modulates *fliC* expression post-transcriptionally and FliW antagonizes CsrA to regulate *fliC* expression through inhibiting CsrA-mediated negative post-transcriptional regulation, indicating a similar partner-switching mechanism “FliW-CsrA-FliC” in *C. difficile*. In *B. subtilis*, two CsrA binding sites (BS1: A51 to A55; BS2: C75 to G82) were identified in the *hag* leader of the mRNA (Yakhnin et al., 2007). Based on the *hag* 5’-UTR sequence and CsrA conserved binding sequence, a 91bp 5’-UTR structure with two potential CsrA binding sites (BS1: 5’-TGACAAGGATGT-3′, BS2: 5’-CTAAGGAGGG-3′) of *fliC* gene was predicted (Supplementary Figure 6; Dubey et al., 2005). Recently, it was also reported that cytoplasmic Hag levels play a central role in maintaining proper intracellular architecture, and the Hag-FliW-CsrA^dimer^ system works at nearly 1:1:1 stoichiometry in *B. subtilis* (Oshiro et al., 2019). Further studies on the exquisite interactions of CsrA, FliW, and *fliC*/FliC in *C. difficile* are still needed.

Flagella play multiple roles in bacterial motility, colonization, growth, toxin production, and survival optimization (Harshey, 2003; Duan et al., 2013; Stevenson et al., 2015). Recently, several papers have reported that the flagellar genes can affect toxin expression in *C. difficile*, but results from different research groups were controversial (Aubry et al., 2012; Baban et al., 2013; Stevenson et al., 2015). Aubry et al. (2012) reported that disruption of some early-stage flagellar genes (F3), such as *fliF*, *fliG*, and *fliM*, could lead to a significant reduction in *tcdR*, *tcdE*, *tcdA*, and *tcdB* expression in *C. difficile* 630Δ*erm*, but no significant difference of *tcdC* expression was detected. Inversely, disruption of late-stage flagellar genes (F1) such as *fliC* increased toxin expression in *C. difficile* 630Δ*erm*. In 2013, Baban et al. (2013) reported that the mutation of *flgE* (one of the F3 genes) resulted in a tenfold reduction in *tcdA* expression and corroborated that the expression of *tcdA* in a *fliC* mutant increased 44.4-fold compared to the wild-type strain *C. difficile* 630Δ*erm*. Surprisingly, Aubry et al. (2012) found that a glycosylation gene (CD0240, one of F2 region genes) mutation, which can totally abolish *C. difficile* 630 motility, but did not change toxin expression. Meanwhile, cyclic diguanylate (C-di-GMP), a cellular second messenger, was also reported to be involved in bacterial motility, biofilm formation, and toxin production by repressing the expression of flagellar genes in *C. difficile* (Purcell et al., 2012; McKee et al., 2013). While we did not detect the C-di-GMP concentration in *C. difficile*, it could be perturbed by *fliW* and *csrA* deletion affecting *C. difficile* physiology. It was hypothesized that the regulation of the flagellar genes on toxin expression could be caused by the direct change or loss of flagellar genes (such as *fliC* gene deletion) rather than loss of the functional flagella (Stevenson et al., 2015). Future study about *fliC* deletion in M120 will be very interesting and will further address the *fliC* gene function in *C. difficile* as there are no flagella in RT078 strains. In our study, data indicate that CsrA negatively modulates *fliC* translation and also plays a positive regulation in toxin expression. Inversely, FliW works against CsrA to regulate *fliC* expression, which can negatively regulate toxin production. While studies of flagellar effects on motility and toxin production in *C. difficile* from different groups were controversial, the role of the flagella in *C. difficile* pathogenicity cannot be overlooked. Dingle et al. (2011) and Baban et al. (2013) both showed higher mortality of the *fliC* mutant in the animal model of CDI compared to the wild-type strains. Our study showed results similar to the published data suggesting that R20291ΔW whose FliC production was dramatically suppressed exhibited higher fatality, while R20291ΔWA showed a decreased pathogenicity compared to R20291 (Figure 6). In 2014, Barketi-Klai et al. (2014) examined the pleiotropic roles of the *fliC* gene in R20291 during colonization in mice. Interestingly, the transcription of *fliW* and *csrA* in the *fliC* mutant was 2.03- and 4.36-fold, respectively, of that in R20291 *in vivo* experiment (Barketi-Klai et al., 2014), which further corroborated that there is a coregulation among *fliC*, *fliW*, and *csrA*. Surprisingly, transcription of *treA*, a trehalose-6-phosphate hydrolase, increased 177.63-fold in the *fliC* mutant compared to that of R20291 during *in vivo* infection (Barketi-Klai et al., 2014). Recently, Collins et al. (2018) hypothesized that dietary trehalose can contribute to the virulence of epidemic *C. difficile*. The relationship of FliW, CsrA, FliC, and trehalose metabolization is another interesting question in *C. difficile*, and some other carbon metabolism affected by the *fliC* mutation could also facilitate *C. difficile* pathogenesis *in vivo*. Previous studies have also highlighted that the flagella of *C. difficile* play an important role in toxin production, biofilm formation, and bacterial adherence to the host (Tasteyre et al., 2001; Dingle et al., 2011; Aubry et al., 2012; Baban et al., 2013; Ethapa et al., 2013). In this study, we showed that the FliW defect led to a significant motility decrease, while the biofilm, adhesion, and toxin production increased significantly. Inversely, R20291ΔWA-W, which can imitate the *csrA* gene deletion, showed an increase in motility and a decrease in biofilm formation, toxin production, and adhesion.

In conclusion, we characterized the function of FliW and CsrA and showed the pleiotropic functions of FliW and CsrA in R20291. Our data suggest that *fliW* and *csrA* play important roles in flagellin (FliC) synthesis, which could contribute to *C. difficile* pathogenicity. Currently, *in vitro* study of the interactions of CsrA, FliW, and *fliC*/FliC in *C. difficile* is underway in our group.

## Data Availability Statement

The original contributions presented in the study are included in the article/Supplementary Material, further inquiries can be directed to the corresponding author.

## Ethics Statement

The animal study was reviewed and approved by the Institutional Animal Care and Use Committee of University of South Florida.

## Author Contributions

DZ and XS designed the experiments. DZ wrote the manuscript. DZ and SW performed the experiments. DZ and XS revised the manuscript. All authors contributed to the article and approved the submitted version.

## Funding

This work was supported in part by the National Institutes of Health grants (R01-AI132711 and R01-AI149852).

## Conflict of Interest

The authors declare that the research was conducted in the absence of any commercial or financial relationships that could be construed as a potential conflict of interest.

## Publisher’s Note

All claims expressed in this article are solely those of the authors and do not necessarily represent those of their affiliated organizations, or those of the publisher, the editors and the reviewers. Any product that may be evaluated in this article, or claim that may be made by its manufacturer, is not guaranteed or endorsed by the publisher.

## Footnotes

^1^ http://big.hanyang.ac.kr/cindel/

^2^ http://www.rgenome.net/cas-offinder/

^3^ https://img.jgi.doe.gov/
