# Supplementary material for: FliW and CsrA Govern Flagellin (FliC) Synthesis and Play Pleiotropic Roles in Virulence and Physiology of Clostridioides difficile R20291
Source: Front Microbiol. 2021 Oct 5;12:735616. doi: 10.3389/fmicb.2021.735616 (PMC8523840; doi:10.3389/fmicb.2021.735616)
Supplement: Supplementary file 1 [file Data_Sheet_1.PDF]

Supplementary Figures and Tables

Supplementary Figures

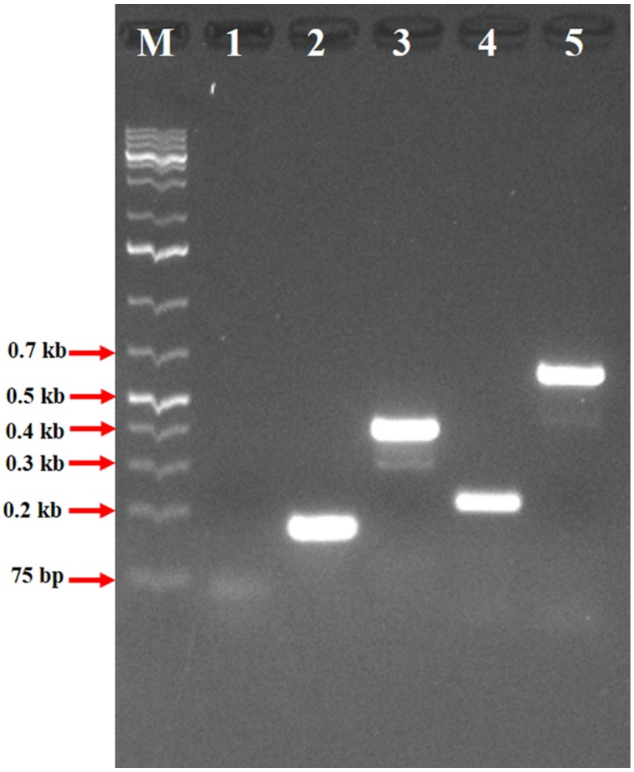

Supplementary Figure 1. Identification of *fliW-csrA* cotranscription by using RT-PCR

M: DNA ladder. 1: 16s primer PCR test with isolated total mRNA. 2: 16s primer PCR test with R20291 cDNA. 3: *fliW* primer 3-F/R PCR test with R20291 cDNA. 4: *csrA* primer 4-F/R PCR test with R20291 cDNA. 5: *fliW-csrA* primer 5-F/R PCR test with R20291 cDNA.

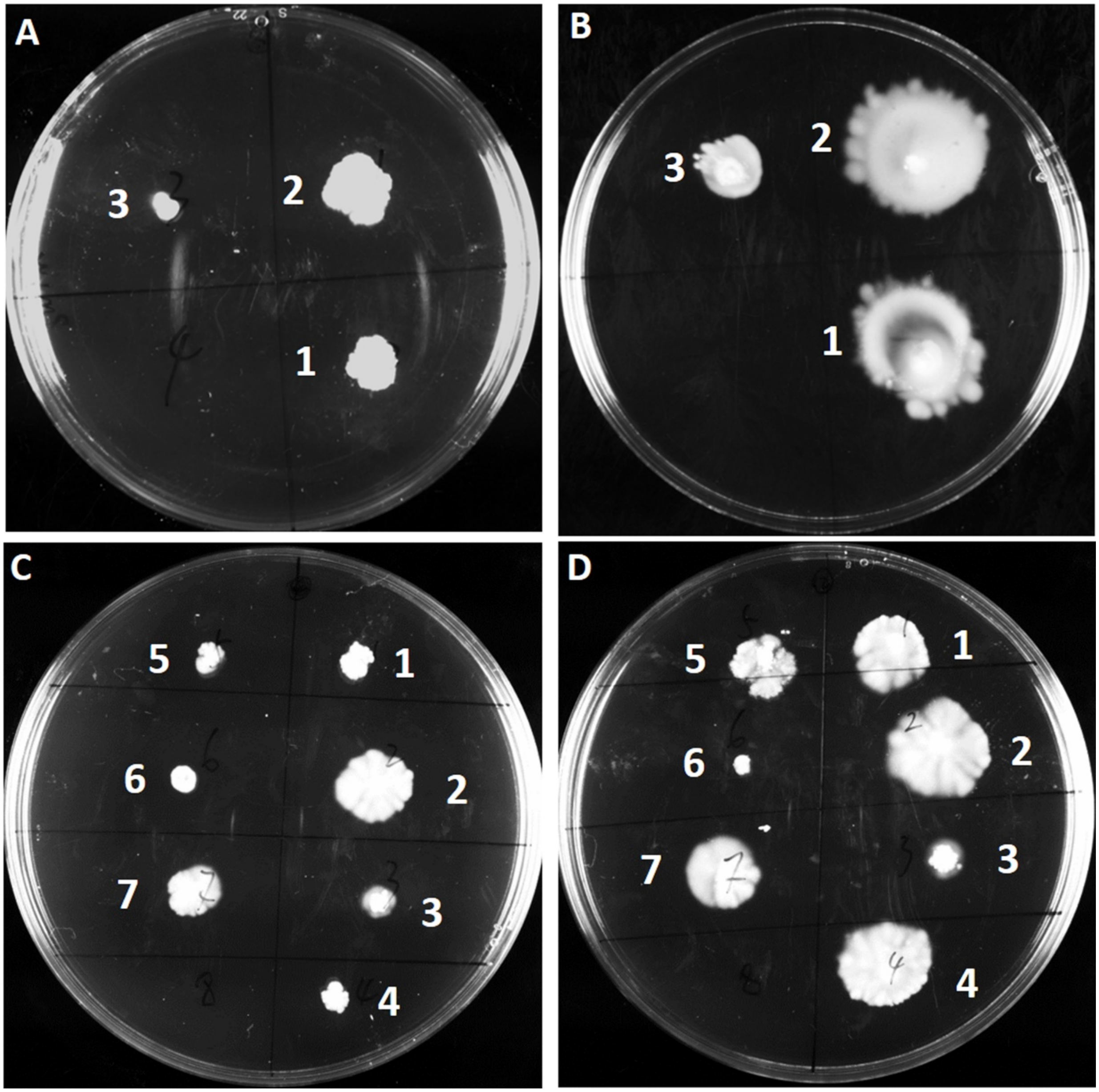

Supplementary Figure 2. Motility analysis and halo diameter

(A) Swarming analysis (0.3% agar plate). 1: R20291 (8.5 ± 1.4 mm); 2: R20291ΔWA (10.8 ± 1.3*); 3:R20291ΔW (4.8 ± 0.6*). (B) Swimming analysis (0.175% agar plate). 1: R20291 (17.2 ± 2.2); 2: R20291ΔWA (25.6 ± 1.7*); 3:R20291ΔW (10.1 ± 1.8*). (C) Swarming analysis. 1: R20291-E (7.5 ± 0.8); 2: R20291ΔWA-E (10.5 ± 1.7*); 3: R20291ΔWA-A (3.5 ± 0.6*); 4: R20291ΔWA-W (7.5 ± 1.7); 5: R20291ΔWA-WA (6.7 ± 1.3); 6: R20291ΔW-E (4.2 ± 0.5*); 7: R20291ΔW-W (8.3 ± 1.3). (D) Swimming analysis. 1: R20291-E (16.3 ± 3.0); 2: R20291ΔWA-E (25.4 ± 3.8*); 3: R20291ΔWA-A (6.2 ± 2.0*); 4: R20291ΔWA-W (15.3 ± 3.2); 5: R20291ΔWA-WA (13.2 ± 1.0); 6: R20291ΔW-E (5.6 ± 1.5*); 7: R20291ΔW-W (17.7 ± 2.6). Complementation plasmid pMTL84153, pMTL84153-*fliW*, pMTL84153-*csrA*, and pMTL84153-*fliW-csrA*, were shorted as E, W, A and WA, respectively. Experiments were independently repeated thrice. Bars

stand for mean ± SEM (*$P<0.05$). One-way ANOVA with post-hoc Tukey test was used for statistical significance.

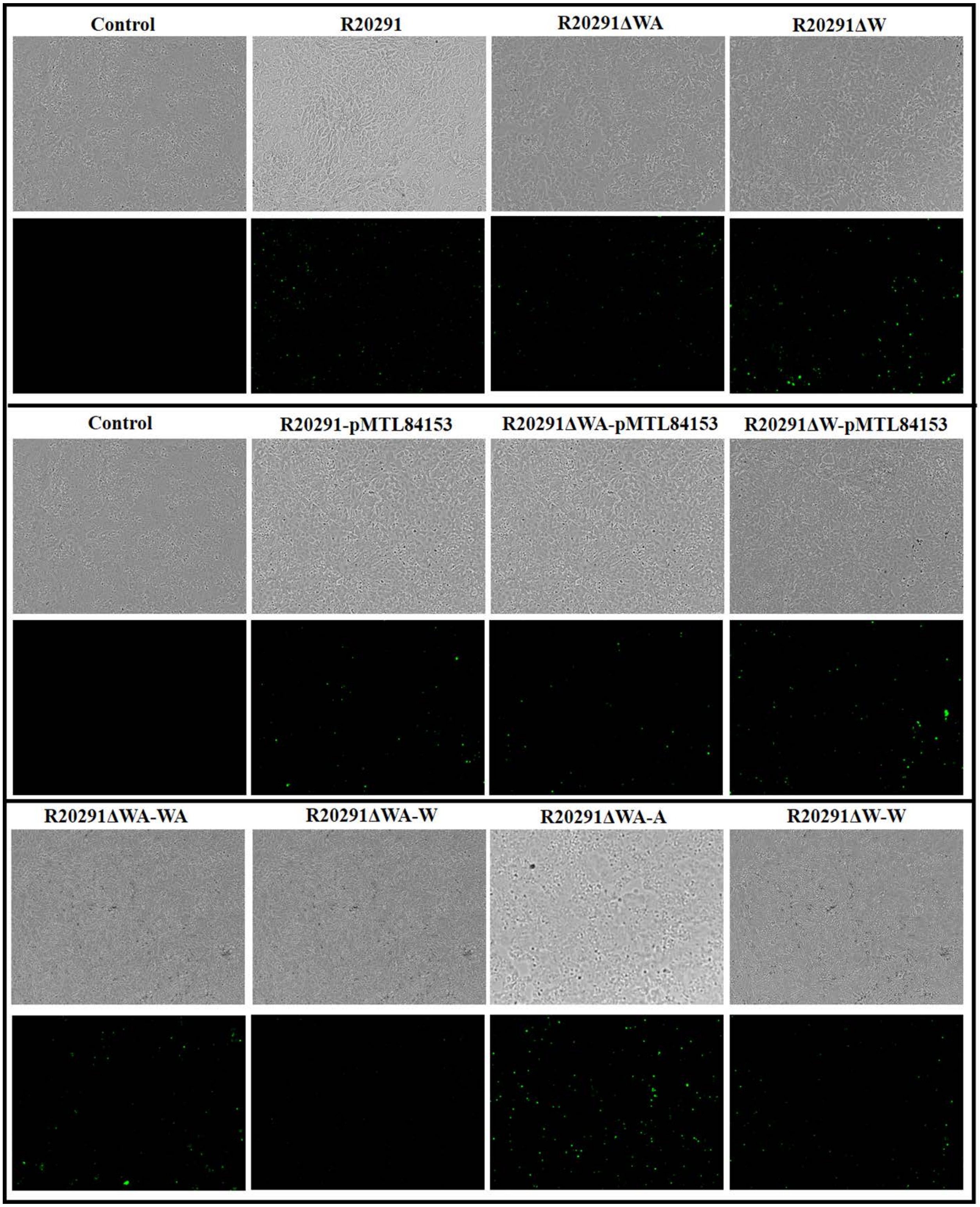

Supplementary Figure 3. Visualization of *C. difficile* adhered to HCT-8 cells

C. difficile vegetative cells were labeled with the chemical 5(6)-CFDA (5-(and-6)-Carboxyfluorescein diacetate) and detected by a Fluorescence Microscope for adhesion analysis. Up channel was the bright field, and down channel was the green field.

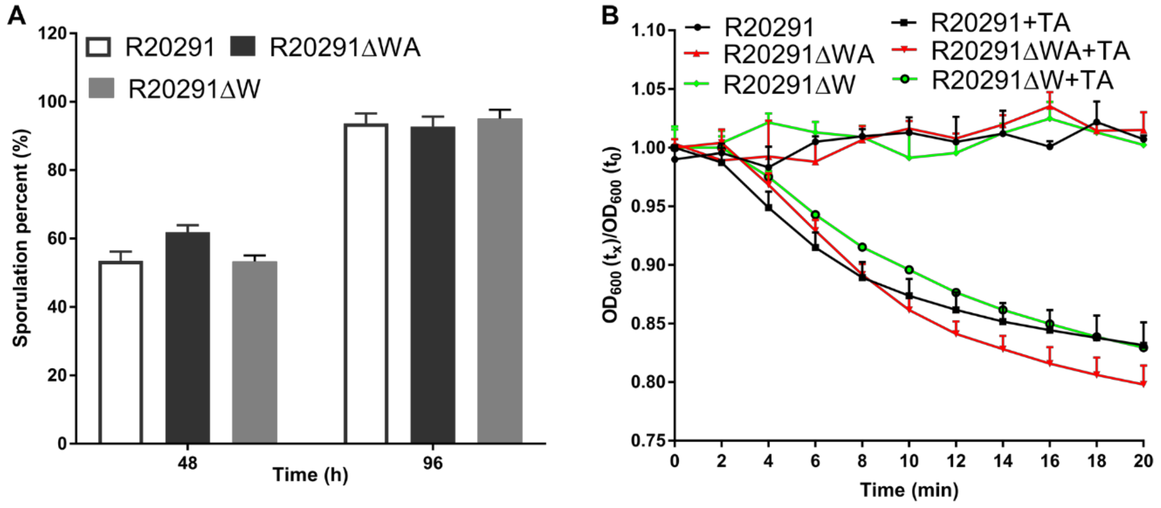

Supplementary Figure 4. Sporulation and germination assay

(A) Sporulation assay. (B) Germination assay. Spores in germination buffer without TA were used as the negative control. Experiments were independently repeated thrice. One-way ANOVA with post-hoc Tukey test was used for statistical significance.

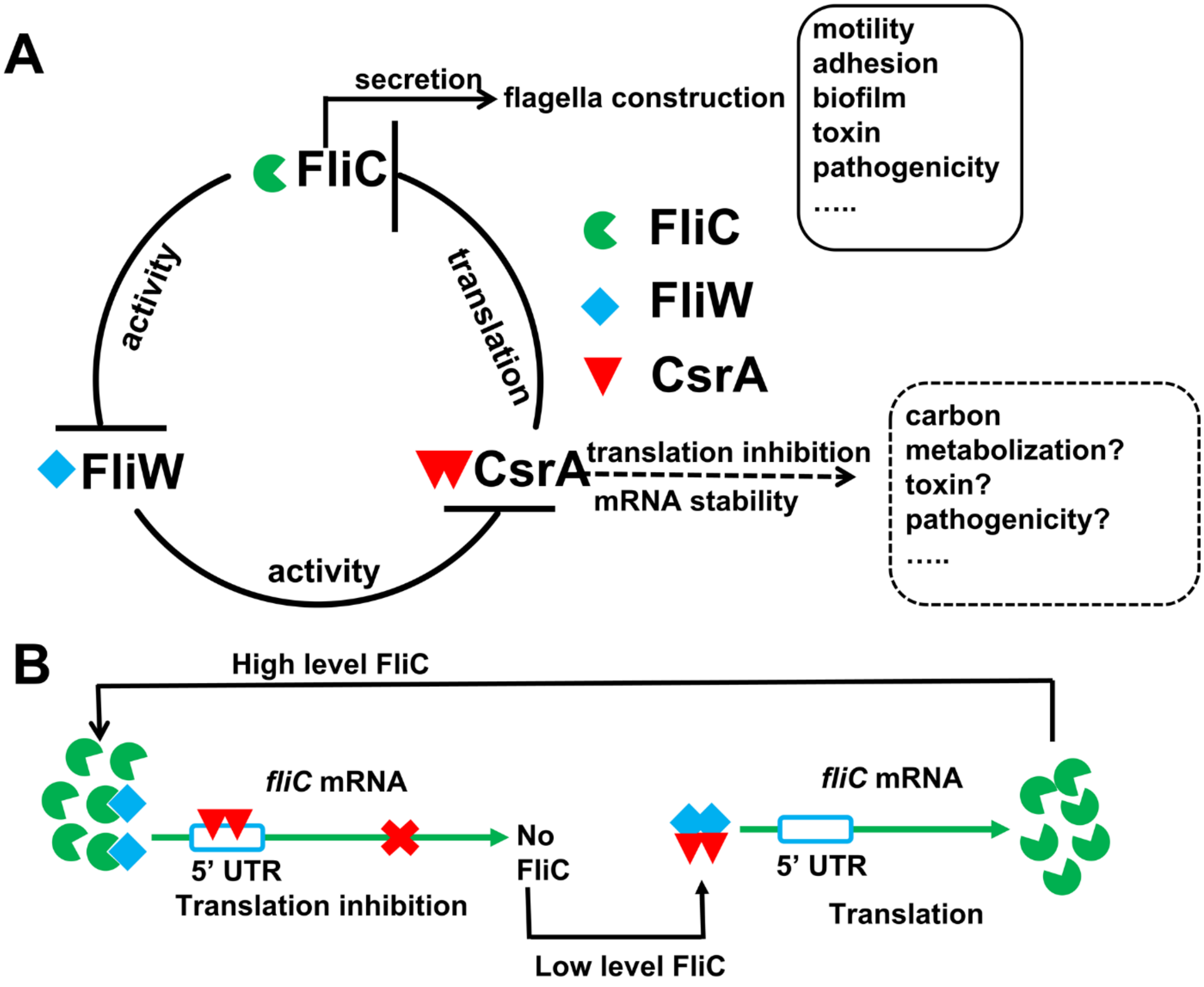

Supplementary Figure 5. Predicted partner-switching mechanism (FliC-FliW-CsrA^{dimer}) in *C. difficile*

Figure S5 was drawn based on an earlier report (1).

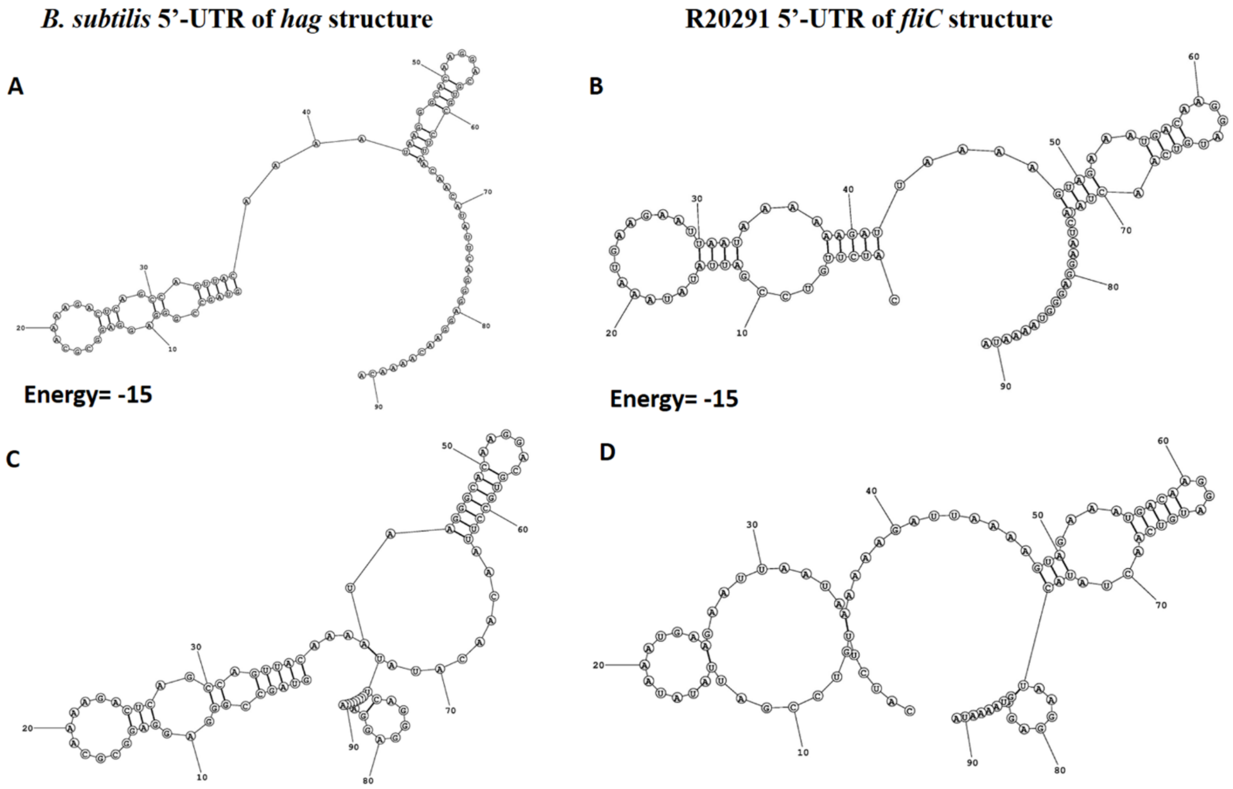

Supplementary Figure 6. Prediction of 5'-UTR structure of *fliC* gene in *C. difficile* R20291

(A) and (C) *B. subtilis* 5'-UTR of *hag* structure, (B) and (D) R20291 5'-UTR of *fliC* structure. (A) and (B) RNAstructure **dynalign** Results (Calculate the lowest free energy secondary structures common to two unaligned sequences); (C) and (D) RNAstructure **PARTS** Results (Predict the common secondary structure, including base pair probabilities, for two unaligned sequences)

Supplementary Tables

Supplementary Table 1. Primers utilized in this study.

Primer	Sequence (5' to 3')
WA(W)-gRNA-F	AAAGTTAAAAGAAGAAAATAGAAATATAATCTTTAATTTGAAAAGATTTA
WA(W)-gRNA-R	TTTCATGTTCACAGTTGATTCTTATCTACAAGAGTAGAAATTAATGGT
WA(W)-Up-F	ATAAGAATCAACTGTGAACATGAAATAATTTCTACTCTTGTAGATACGGTTCAGTGAGCTTAAAGT
WA(W)-Up-R	ATGTAACCTTCATCATATCTGCG
WA-Down-F	AACGCAGATATGATGAAGGTTACATGATGGAAACAATGTGAAACTAGC
WA-Down-R	CATGCTGATCTAGATTTCTCCATAGCAGCTAAATTTAATTCCATTATTTG
W-Down-F	AACGCAGATATGATGAAGGTTACATTAAAAACTAATTTTGGAATGCAAAT
W-Down-R	CATGCTGATCTAGATTTCTCCATAG TTGCCCATTGTCCTGCAT
1-C-F	AGGTGTACCACCTGTTGAAT
1-C-R	TTGCCCATTGTCCTGCAT
2-C-R	CAGCTAAATTTAATTCCATTATTTG
3-F	ATGACCATGATTACGAATTCGAGCTATGATGAAGGTTACATTAAAAAAAGG
3-R	CGCGTGACGTCGACTCTAGAGGATCTTACTAGCATCCATTATCACC
4-F	ATGACCATGATTACGAATTCGAGCTATGCTAGTAATTTCAAGAAAAAAAGATGAAGC
4-R	CGCGTGACGTCGACTCTAGAGGATCTTATTTTAATGACTTTAAAATTTTTAT
5-F	ATGACCATGATTACGAATTCGAGCTATGATGAAGGTTACATTAAAAAAAGG
5-R	CGCGTGACGTCGACTCTAGAGGATCTTATTTTAATGACTTTAAAATTTTTAT
Q-*tcdA*-F	GCGGAAATGGTAGAAATG
Q-*tcdA*-R	ATCAGGTGCTATCAATACTT
Q-*tcdB*-F	GTATTACCTAATGCTCCAA
Q-*tcdB*-R	CACCTTCATAGTTATCTCTT
Q-*fliC*-F	AGAGATACAGATGTTGCTTCA
Q- *fliC*-R	TCCTTGTGGTTGCTGATTA
16s-F	CCGTAGTAAGCTCTTGAA
16s-R	TGGTGTTCCTCCTAATATC

Supplementary material references

1. Mukherjee S, Yakhnin H, Kysela D, Sokoloski J, Babitzke P, Kearns DB. 2011. CsrA-FliW interaction governs flagellin homeostasis and a checkpoint on flagellar morphogenesis in Bacillus subtilis. Mol Microbiol 82:447-61.
